# Spatial and temporal development of deltamethrin resistance in malaria vectors of the *Anopheles gambiae* complex from North Cameroon

**DOI:** 10.1371/journal.pone.0212024

**Published:** 2019-02-19

**Authors:** Stanislas Elysée Mandeng, Herman Parfait Awono-Ambene, Jude D. Bigoga, Wolfgang Eyisap Ekoko, Jérome Binyang, Michael Piameu, Lili Ranaise Mbakop, Betrand Nono Fesuh, Narcisse Mvondo, Raymond Tabue, Philippe Nwane, Rémy Mimpfoundi, Jean Claude Toto, Immo Kleinschmidt, Tessa Bellamy Knox, Abraham Peter Mnzava, Martin James Donnelly, Etienne Fondjo, Josiane Etang

**Affiliations:** 1 Institut de Recherche de Yaoundé (IRY), Organisation de Coordination pour la lutte contre les Endémies en Afrique Centrale (OCEAC), Yaoundé, Cameroon; 2 Laboratory of General Biology, University of Yaounde I, Yaounde, Cameroon; 3 Laboratory for Vector Biology and control, National Reference Unit for Vector Control, The Biotechnology Center, Nkolbisson-University of Yaounde I, Yaounde, Cameroon; 4 Laboratory of Animal Biology and Physiology, Faculty of Science, University of Douala, Douala, Cameroon; 5 Ecole des Sciences de la Santé, Université Catholique d’Afrique Centrale, Yaoundé, Cameroon; 6 National Advanced School of Engineering, University of Yaounde I, Yaounde, Cameroon; 7 Ministry of Public Health, National Malaria Control Programme, Yaounde, Cameroon; 8 Department of Infectious Disease Epidemiology, London School of Tropical Medicine & Hygiene, MRC Tropical Epidemiology Group, London, United Kingdom; 9 School of Public Health, University of the Witwatersrand, Johannesburg, South Africa; 10 Global Malaria Programme, World Health Organization, Geneva, Switzerland; 11 African Leaders Malaria Alliance (ALMA), Dar es Salaam, Tanzania; 12 Department of Vector Biology, Liverpool School of Tropical Medicine, Liverpool, United Kingdom; 13 Department of biological sciences, Faculty of Medicine and Pharmaceutical Sciences, University of Douala, Douala, Cameroon; 14 Institute for Insect Biotechnology, Justus Liebig University Gießen, Heinrich-Buff-Ring, Germany; Arizona State University, UNITED STATES

## Abstract

The effectiveness of insecticide-based malaria vector control interventions in Africa is threatened by the spread and intensification of pyrethroid resistance in targeted mosquito populations. The present study aimed at investigating the temporal and spatial dynamics of deltamethrin resistance in *An*. *gambiae s*.*l*. populations from North Cameroon. Mosquito larvae were collected from 24 settings of the Garoua, Pitoa and Mayo Oulo Health Districts (HDs) from 2011 to 2015. Two to five days old female *An*. *gambiae s*.*l*. emerging from larval collections were tested for deltamethrin resistance using the World Health Organization’s (WHO) standard protocol. Sub samples of test mosquitoes were identified to species using PCR-RFLP and genotyped for knockdown resistance alleles (*Kdr* 1014F and 1014S) using Hot Ligation Oligonucleotide Assay (HOLA). All the tested mosquitoes were identified as belonging to the *An*. *gambiae* complex, including 3 sibling species mostly represented by *Anopheles arabiensis* (67.6%), followed by *Anopheles coluzzii* (25.4%) and *Anopheles gambiae* (7%). Deltamethrin resistance frequencies increased significantly between 2011 and 2015, with mosquito mortality rates declining from 70–85% to 49–73% in the three HDs (Jonckheere-Terstra test statistic (*JT)* = 5638, *P*< 0.001), although a temporary increase of mortality rates (91–97%) was seen in the Pitoa and Mayo Oulo HDs in 2012. Overall, confirmed resistance emerged in 10 *An*. *gambiae s*.*l*. populations over the 24 field populations monitored during the study period, from 2011 to 2015. Phenotypic resistance was mostly found in urban settings compared with semi-urban and rural settings (*JT* = 5282, *P*< 0.0001), with a spatial autocorrelation between neighboring localities. The *Kdr* 1014F allelic frequencies in study HDs increased from 0–30% in 2011 to 18–61% in 2014–2015 (*JT* = 620, *P* <0.001), especially in *An*. *coluzzii* samples. The overall frequency of the *Kdr* 1014S allele was 0.1%. This study revealed a rapid increase and widespread deltamethrin resistance frequency as well as *Kdr* 1014F allelic frequencies in *An*. *gambiae s*.*l*. populations over time, emphasizing the urgent need for vector surveillance and insecticide resistance management strategies in Cameroon.

## Introduction

Malaria is one of the most dangerous parasitic diseases of the current decade [1] impacting on the health and standard of living of populations in endemic areas. Despite huge efforts conceded for its elimination, about 3.2 billion people living in 97 countries worldwide are still at risk, especially children under 5 years living in sub-Saharan Africa [2]. Since no antimalarial vaccine is commercially available, efforts to prevent the disease rely mainly on vector control tools such as long-lasting insecticidal nets (LLINs) and indoor residual spraying (IRS) [3]. However, these measures are threatened by rapid expansion of insecticide resistance in vector populations [4, 5]. Insecticidal products belonging to five chemical classes (carbamates, neonicotinoids, organochlorines, organophosphates, and pyrethroids) are recommended or prequalified by the World Health Organization for adult malaria vector control [6]. For LLINs, the range of available insecticides is very limited. Currently, only three pyrethroid insecticides (deltamethrin, permethrin and alpha-cypermethrin) are used in prequalified LLIN products, because of their low toxicity to mammals, fast action, and high residual insecticidal activity [7]. Unfortunately, resistance particularly to DDT and pyrethroids is spreading widely in the major malaria vector species, *An*. *gambiae s*.*l*. and *Anopheles funestus* group [8, 9]. Extensive use of similar insecticides in both agriculture and public health including scaling up insecticide treated bed nets during the past decades is considered to have induced the emergence and rapid expansion of insecticide resistance [10, 11, 12]. Two main physiological mechanisms confer insecticide resistance in malaria vectors: target site modification including mutations at the voltage gate sodium channel (*Kdr* 1014F or 1014S mutations) conferring resistance to DDT and pyrethroids, and cetylcholinesterase (G119S *Ace-1* mutation) conferring resistance organophosphates and carbamates [13, 14, 15], and detoxification enzymes involved in insecticide degradation [16, 17]. Both leucine to phenylalanine (L1014F) and leucine to serine (L1014S) substitutions are known to decrease affinity of pyrethroids for the receptor of voltage gate sodium channel [13]. Metabolic resistance operates through higher catalytic properties and/or overexpression of detoxification genes such carboxylesterases (CoEs), cytochrome P450 mono-oxygenases (P450s) and glutathione S-transferases (GSTs) [18]. Furthermore, it is now common to find mosquitoes displaying multiple insecticide resistance mechanisms [19].

In Cameroon, malaria causes more than 30% of annual deaths in hospitals and health facilities, 30% of morbidity cases, 36% of consultations and 48% of hospitalizations annually [20]. Since 2000 the National Malaria Control Programme (NMCP) has implemented the distribution of impregnated bed nets. In 2011, more than 8 million LLINs were freely distributed throughout the country. This initiative was reinforced by distributing over 12 million LLINs that are recommended by the WHO Pesticide Evaluation Scheme (WHOPES), between 2015 and 2016. LLINs distributed in 2011 were mainly the PermaNet 2.0 mosquito (0.055 g/m^2^ deltamethrin coated on polyester, Vestergaard, Lausanne, Switzerland) whereas those distributed in 2015 were either PermaNet 2.0 or Olyset nets (1 g/ m^2^ permethrin incorporated into polyethylene, Sumitomo Chemical, Tokyo, Japan), or Interceptor (A polymer binder system combined with 0.2 g/m^2^ alpha-cypermethrin, BASF chemical company, Ludwigshafen, Germany).

Studies conducted so far across the country reported an increase in the prevalence of insecticide resistance and heterogeneous patterns in vector populations [19, 21]. While in the southern part of the country, high prevalence of insecticide resistance is associated positively with high prevalence of *Kdr* 1014 alleles, in the northern parts, moderate resistance, mainly associated with metabolic resistance was reported in cotton growing areas [21, 22, 23]. However, little is known about the dynamics of insecticide resistance in malaria vectors from different ecological settings, particularly between urban and remote rural settings.

Therefore, this study sought to assess the spatial and temporal distribution of deltamethrin resistance in wild populations of the *An*. *gambiae* complex in 24 sites across northern Region of Cameroon, after LLINs distribution in 2011.

## Materials and methods

### Study sites

**Fig 1:**
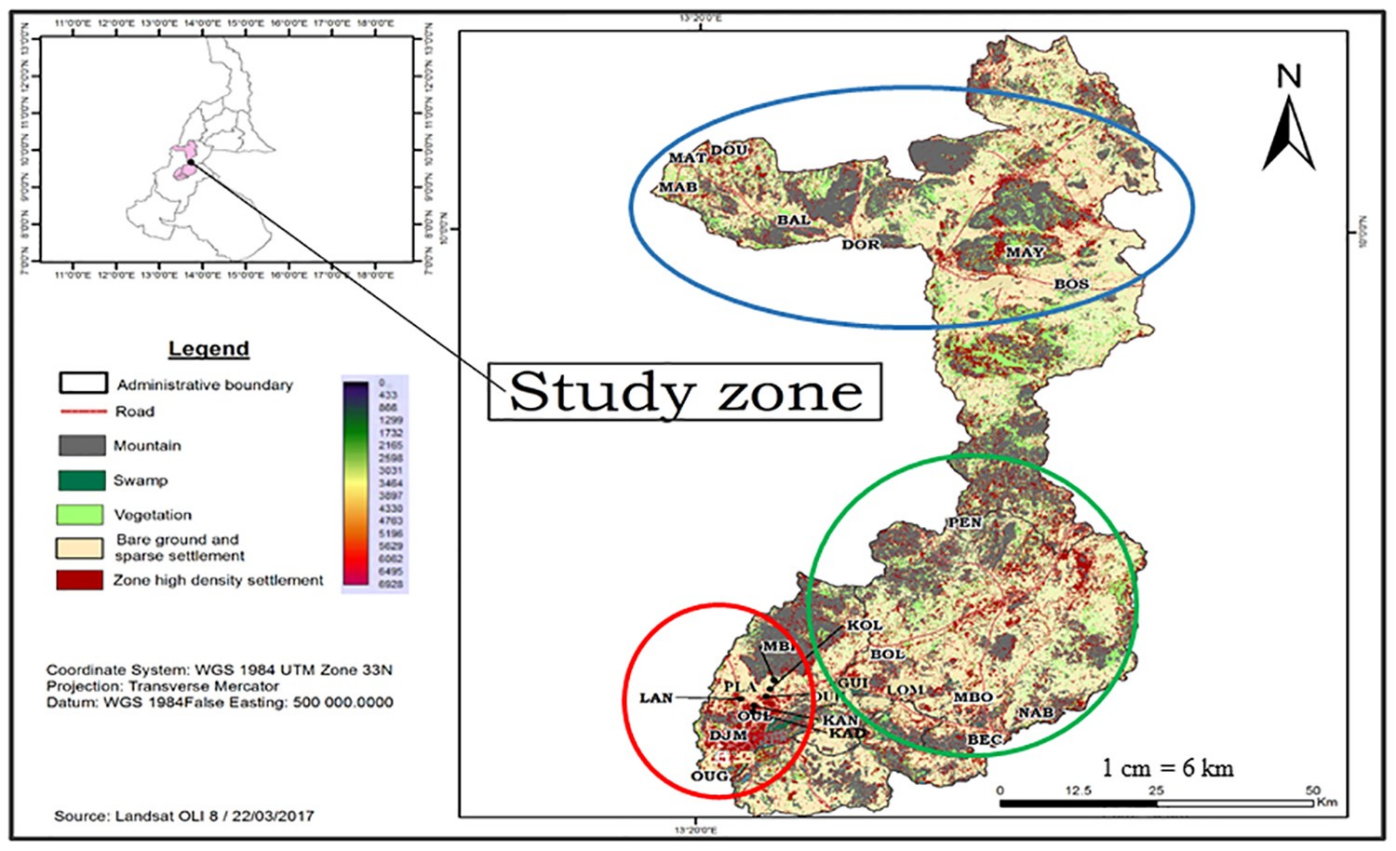
Map of North Cameroon showing study Health Districts (HDs) and clusters. The red chart encloses cluster of the Garoua HD (LAN: Lainde II; OUL: Ouro lawane; PLA: Plateau; DJM: Djamboutou; OUG: Ourogarga; KAN: Kanadi I; KAD: Kanadi II; MBI: Mbilga; OUH: Ouro housso; KOL: Kollere). The green chart encloses cluster of the Pitoa HD (PEN: Pene; BOL: Boulgou; LOM: Lombou; MBO: Mbolom; NAB: Nassarao-be; BEC: Be-centre; GUI: Guizigare). The Blue chart encloses cluster of the Mayo Oulo HD (DOU: Doumou; BOS: Bossoum; DOR: Dourbeye; MAY: Mayo oulo; MAB: Maboni; MAT: Matra; BAL: Bala).

The study was conducted in 24 locations within 3 Health Districts (HD) of the Northern region of Cameroon; namely Garoua (9°18'N; 13°24'E) the capital city of the North Region, Mayo Oulo (9°46'N; 13°44'E) located 90 km to the north of Garoua, and Pitoa (9°24'N; 13°31'E) located 18 km north-East of Garoua (Fig 1). These HDs are located along the Bénoué River basin in humid tropical domain. The climate in these areas is characterized by two seasons: one dry season extending from November to May and one rainy season from June to October, with average annual rainfall between 700–1000 mm and a mean annual temperature of 28.1°C [24]. Table 1 presents the geo-localization and ecological features of the 24 study locations, including 14 rural, 7 urban and 3 peri-urban settings, classified according to the rates of built-up land (RBL), i.e.: RBL>50% for urban areas, RBL<40% for rural areas; 40< RBL<50 for peri-urban areas proximate to the city.

**Table 1:** Study sites and their ecological features.

Health Districts	Population of District	Locality	Geographical coordinates	Ecological features	Rate of built-up land (% RBL)
**Garoua**	316 957	Kanadi II*	09°18’05,9”N -13°22’09,6”E	urban	85
		Kanadi I*	09°18’07,7”N-13°22’29,9”E	urban	80
		Ouro housso*	09°17’35,4”N-13°22’37,8”E	urban	82
		Lainde II	09°20’39,7”N-13°25’01,5”E	urban	87
		Djamboutou II*	09°18’19,3”N-13°20’48,3”E	urban	79
		Plateau	09°18’21,3”N-13°22’55,1”E	urban	83
		Ouro lawane	09°21’30,7”N-13°25’39,4”E	peri-urban	45
		Mbilga*	09°20’40,8”N-13°23’04,2”E	peri-urban	40
		Kollere*	09°18’02,9”N-13°23’40,2”E	urban	78
		Ouro garga*	09°16’30,4”N-13°17’55,7”E	rural	20
**Pitoa**	108 611	Guizigare*	09°24’25,2”N-13°31’01,8”E	peri-urban	47
		Pene	09°34’47”N-13°37’13,0”E	rural	15
		Boulgou	09°25’13”N-13°28’87”E	rural	18
		Nassarao-be	09°18’58”N-13°43’22”E	rural	22
		Lombou*	09°20’54”N-13°32’25”E	rural	12
		Be-centre*	09°18’54”N-13°40’21”E	rural	9
		Mbolom	09°19’72”N-13°39’29”E	rural	13
**Mayo Oulo**	91 501	Mayo oulo*	09°58’02,3”N-13°36’81,9”E	rural	25
		Doumou	10°03’646”N-13°18’25,2”E	rural	18
		Matra	10°03’206”N-13°18’15,9”E	rural	20
		Maboni	10°02’75,0”N-13°19’90,7”E	rural	13
		Dourbeye*	09°59’67,2”N-13°30’21,6”E	rural	15
		Bossoum	09°56’22,8”N-13°42’50,6”E	rural	10
		Bala*	10°00’36,7”N-13°28’02,4”E	rural	7

Sites with asterix (*) are those where a complete data set from bio assays to molecular analysis was collected.

### Mosquito collection

Mosquito larvae were sampled in 24 study sites between September and November each year for 5 consecutive years from 2011 to 2015, using the dipping technique [25]. In each locality, anopheline larvae were collected from mosquito breeding sites (temporary pools of water, permanent shelters, hoof prints, rice paddies flooded with water) for testing susceptibility to deltamethrin. No specific permissions were required for larval collection, because the visited breeding sites were outdoors in open access areas. These field studies did not involve endangered or protected species.

Mosquito larvae from the field were brought to local insectaries, fed with TetraMindBaby (fry food), and reared until adult emergence. Adults were fed with 10% glucose solution, and identified using morphological identification reference keys [26, 27]. Only female *An*. *gambiae s*.*l*. were used for bioassays and molecular analyses.

### Insecticide susceptibility bioassays

Bioassays were performed on mosquitoes aged 2–5 days using WHO susceptibility test kits and standard protocol for adults [28] under ambient room temperature ranging from 25°C to 28°C and relative humidity of 70–80%. Filter papers impregnated with 0.05% deltamethrin were supplied by the Vector Research and Control Unit of University Sains Malaysia (Penang, Malaysia). Each complete set of bioassays was performed with five batches of 20–25 unfed females, four batches were exposed to insecticide impregnated filter papers and one batch was exposed to untreated filter paper as control.

During exposure to insecticides, the number of mosquitoes knocked down was recorded at 5 min intervals. After 1 h exposure, mosquitoes were transferred to holding tubes and provided with cotton pads soaked with 10% sugar solution. The mortality rates were determined 24 h post-exposure. All test samples were individually stored desiccated in 1.5-mL micro centrifuge tubes containing silica gel and kept at -20 °C for molecular analyses.

### Species identification and *kdr* genotyping

Total DNA of mosquitoes used as control samples during susceptibility tests was extracted using the CTAB (Cetyl trimethyl ammonium bromide) method [29] and each mosquito species identified using Polymerase Chain Reaction-Restriction Fragment Length Polymorphism (PCR-RFLP) [30]. This method allows simultaneous identification of the species of the *An*. *gambiae* complex. Alleles at the *Kdr* 1014 locus were genotyped using Hot Oligonucleotide Ligation Assay (HOLA) as described by Lynd *et al*. [31]. Based on the limited number of adult mosquitoes from larval collections in some locations, only samples from 13 representative settings (7 in the Garoua HD, 3 in the Mayo Oulo HD and 3 in the Pitoa HD) where breeding sites were mostly productive were selected for species identification and *kdr* genotyping from 2011 to 2014 (Table 1). In 2015, three sentinel sites were selected, i.e. Ouro Housso I, Bala and Be-Centre in the Garoua, Mayo Oulo and Pitoa HDs respectively for follow up.

### Statistical analysis

Susceptibility test data were excluded from the analysis if fewer than 40 mosquitoes were tested. Resistance status was evaluated in each location and each year according to the WHO criteria [28]: mortality rates <90% was indicative of resistance, 90%≥ mortality rates between ≤97% suggested probable resistance to be confirmed, while mortality rates ≥98% indicated susceptibility. The knock-down times for 50% and 95% mosquitoes (Tkd50 and Tkd95) during bioassays were estimated using a log probit model [32] and analyses were performed using the WIN DL (version 2.0, 1999) software. Comparison of mortality rates was performed using the Chi-square test. Estimates of mortality rates and the 95% confidence interval were determined using the software MedCalc V16.8.4. Allelic frequencies at the *Kdr* 1014 locus were calculated using Genepop Online (Version 4.5.1) [33].

Spatial autocorrelation was explored annually in three HDs using Moran’s I statistic to investigate whether mortality measurements were more similar in clusters geographically closer to each other. Box plots were used to show trends or dissimilarities in the distributions of quantitative variables across years, while 95% bootstrap confidence intervals based on 2,000 samples were reported for the means of quantitative variables across years. Trends in mortality and Tkd50 measurements from rural to urban zones, and trends in the distribution of species and *Kdr* 1014F alleles frequencies over time were assess using Jonckheere-Terstra k-sample test of the null that all samples come from a common population against the alternative that there is a trend. Variation in mortality and *Kdr* 1014F alleles frequencies across Health Districts was examined using the Anderson-Darling k-sample test of the null that all samples come from a common population against the alternative that exist differences in distributions. Non-overlapping 95% confidence intervals (CI) or p-values < 0.05 were considered as statistically significant. In order to show the relationship between localities [respectively variables], the quality of the representation of the localities [respectively variables], as well as, the correlation between localities [respectively variables] and the dimensions; multiple factor analysis (MFA) was performed using FactoMineR package. Health district was added as a supplementary variable.

All the analyses were performed using the R 3.5.0 software (R Development Core Team, 2018). R packages used include: kSamples, boot, shapefiles, maps, mapdata, sp, lme4, spdep, maptools, ggplot2, ggmap, geoR, geoRglm, rgdal, shapefiles, RgoogleMaps, spacetime, FactoMineR, plus other basic packages.

## Results

### Spatio-temporal distribution of species of the *Anopheles gambiae* complex

**Fig 2:**
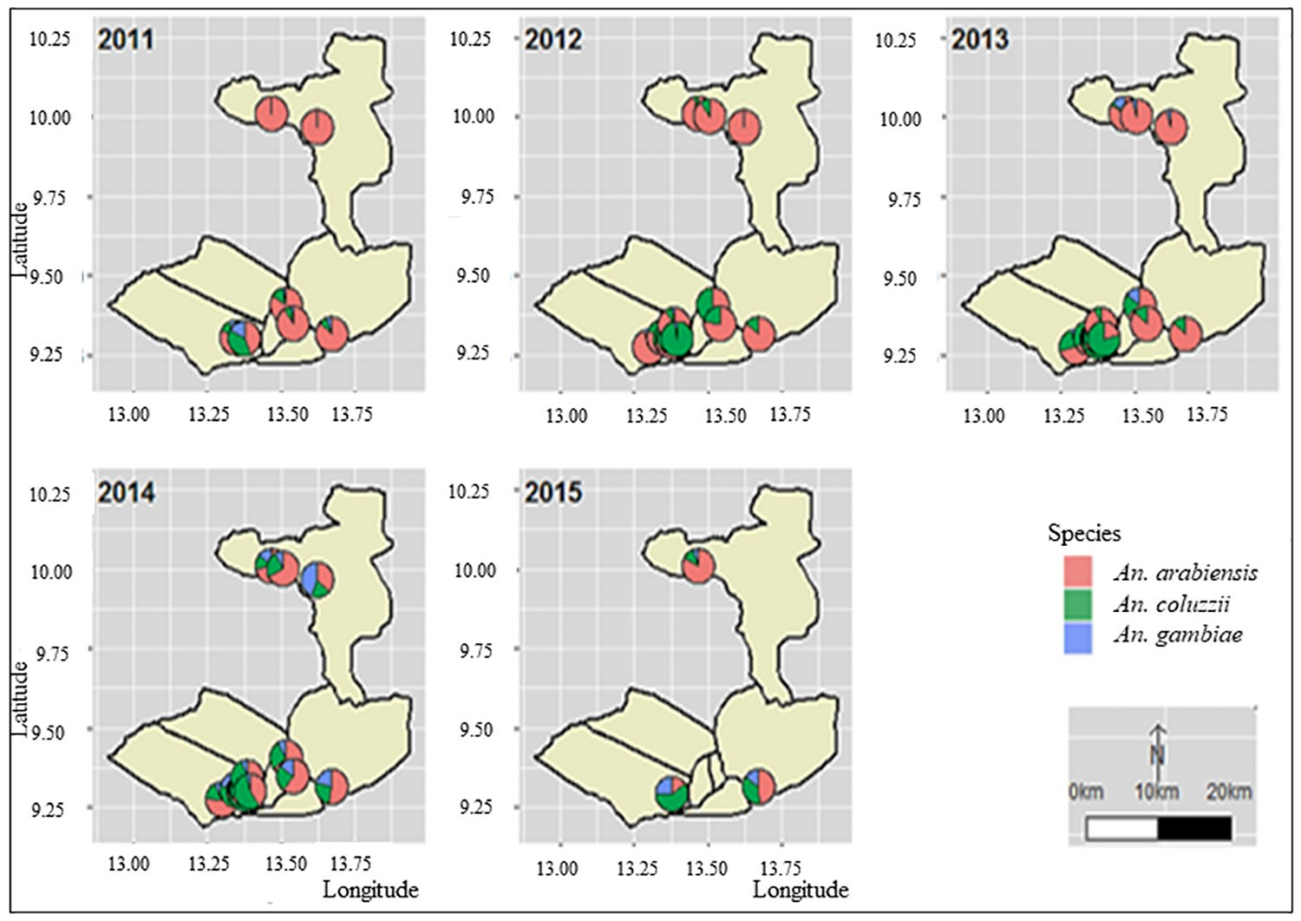
Distribution of species among *An*. *gambiae s*.*l*. samples from 2011 to 2015.

**Fig 3:**
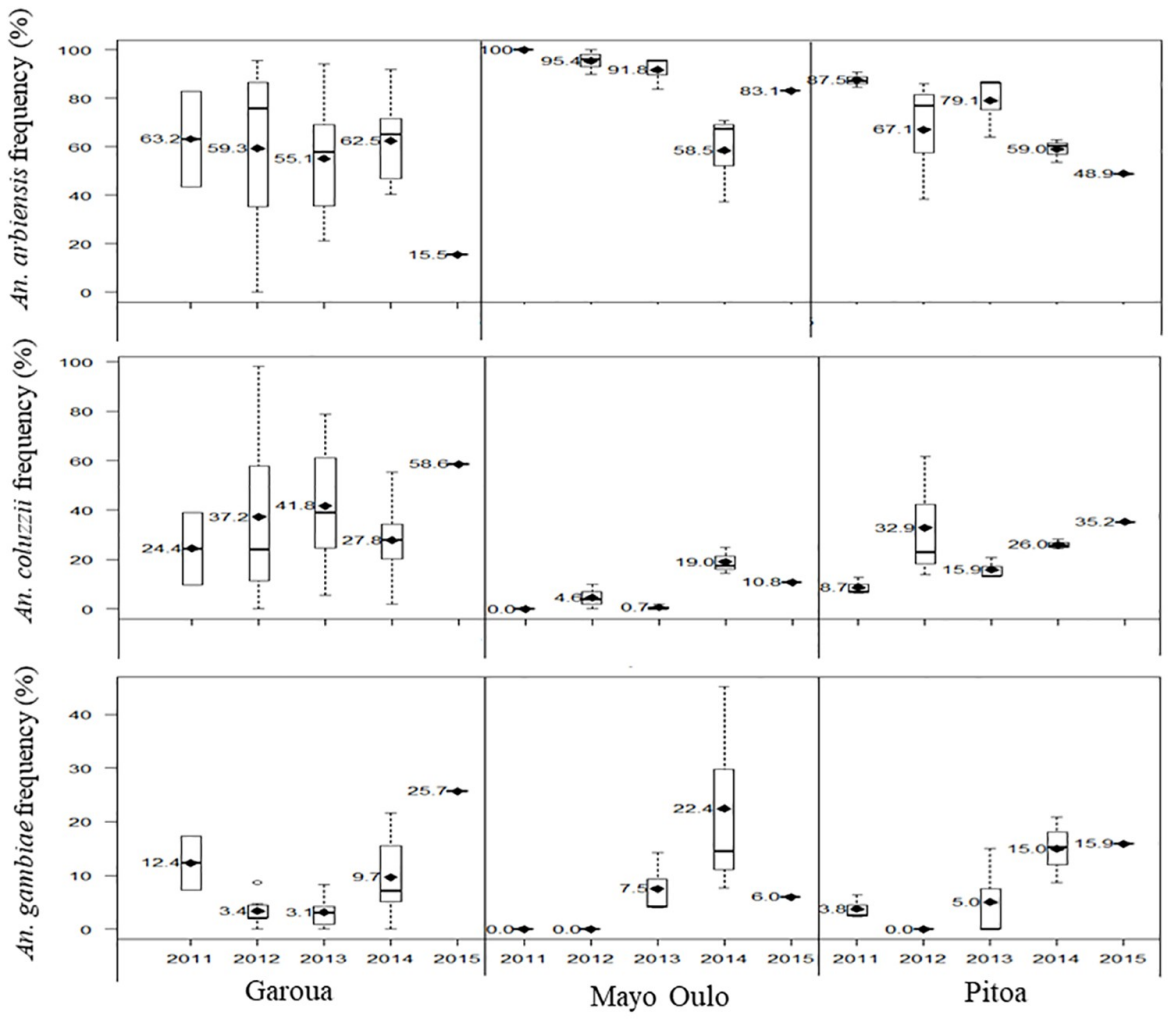
Temporal evolution of *An*. *arabiensis*, *An*. *coluzzii* and *An*. *gambiae* species proportions across the Garoua, Mayo Oulo and Pitoa Health districts (2011–2015).

A total of 2,637 *An*. *gambiae s*.*l*. specimens from 13 representative localities were identified down to the species, including 1,299 specimens from the Garoua HD, 710 specimens from the Pitoa HD and 628 specimens from the Mayo Oulo HD (Table 2). The distribution of the sibling species in the surveyed locations over the 5 years longitudinal survey is presented in Fig 2 and the temporal variations of their proportions across the three HDs in Fig 3. Three species of the *An*. *gambiae* complex were identified; *An*. *arabiensis* was predominant (67.6%), followed *An*. *coluzzii* (25.4%) and *An*. *gambiae* (7%). The three species showed variable spatial and temporal distribution patterns. *An*. *arabiensis* was widespread and present at variable proportions (14–98%) across the 13 study sites and all over the study period. *An*. *coluzzii* which was mostly found in the 10 study sites within the Garoua and Pitoa HDs from 2011 to 2013 (6–98%), was subsequently represented in all the 13 sites in 2014, including the 3 study sites of the Mayo Oulo HD. In some urban settings such as Kollere, Kanadi, Plateau and Ouro housso in the Garoua HD, *An*. *coluzzii* was a major species representing up to 36% of the analyzed samples, while it was less represented in rural settings of the Mayo Oulo HD (7%). From 2011 and 2013, *An*. *gambiae* was found only in 3 study sites among the 13 survey locations, and at very low frequencies (<5%). Subsequently in 2014, this species became widespread across all the study sites, with up to 35% frequencies in few locations (e.g. Mayo Oulo, 2014). In 2015, species distribution in the 3 three selected sentinel sites reflected the heterogeneity observed across the three HDs, i.e.:

- in Ouro Housso I (Garoua HD), there was a predominance of *An*. *coluzzii* (59%) HD, followed by *An*. *gambiae* 26% and *An*. *arabiensis* (15%);
- in Be-centre (Pitoa HD), half of the tested mosquitoes were of *An*. *arabiensis* species (49%), followed by *An*. *coluzzii* (35%) and *An*. *gambiae* (12%);
- in Bala (Mayo Oulo HD), 84% specimens were *An*. *arabiensis* versus 11% and 5% *An*. *coluzzi*i and *An*. *gambiae*. *An*. *gambiae* which was not identified in this site between 2011 and 2012 appeared in 2013, and in two other localities of the Mayo Oulo HD as well.

**Table 2:** Number of *Anopheles gambiae s*.*l*. specimens tested/analysed per health district per year.

Experiment	Health	Number of mosquitoes tested									
	District	2011		2012		2013		2014		2015	
		T	C	T	C	T	C	T	C	T	C
**Susceptibility tests**	Garoua	686	177	996	300	1306	492	1322	565	1331	740
	Pitoa	570	160	794	325	723	172	775	275	1031	273
	Mayo Oulo	315	74	173	50	368	112	534	186	610	138
	**Total**	1,571	411	1,963	675	2,397	776	2,631	1,026	2,972	1,151
**Species identification**	Garoua		87		331		372		451		58
	Pitoa		129		145		157		191		88
	Mayo Oulo		90		151		146		158		83
	**Total**		306		627		675		800		229
***Kdr* genotyping**	Garoua		87		257		252		259		58
	Pitoa		128		110		120		132		88
	Mayo Oulo		90		133		144		134		83
	**Total**		305		500		516		525		229

T: Test mosquitoes (i.e. exposed to deltamethrin impregnated papers); C: Control mosquitoes (i.e. not exposed to deltamethrin impregnated papers)

Considering the overall temporal variations of species proportions in each HD, three different patterns of species distributions were observed (Fig 3). In the Garoua HD, the average frequencies of *An*. *arabiensis* was 55–63% between 2011 and 2014, then it dropped to 15.5% in 2015. Conversely, the frequencies of *An*. *coluzzii* increased from 24% in 2011 to 59% in 2015, although it sporadically declined to 28% in 2014. The frequencies of *An*. *gambiae* showed a parabola-like variation, with the lowest values recorded in 2012–2013 (3%) and the highest in 2011 and 2015 respectively (12% and 26%). In the Mayo Oulo HD, the frequencies of *An*. *arabiensis* was mostly higher than 80% over the years, except in 2014 when it dropped to 58%. The frequencies of *An*. *coluzzii* and *An*. *gambiae* were variable from year to year, i.e. 0% in 2011, increased up to 19–22% between 2012 and 2014, then decreased to 6–11% in 2015. In the Pitoa HD, the frequencies of *An*. *arabiensis* progressively declined from 87% in 2011 to 49% in 2015, while the proportions of *An*. *coluzzii* and *An*. *gambiae* increased from 9% to 35% and from 4% to 16% respectively. However, these variations were nonlinear.

When considering the data from the three HDs together, there was a significant decrease in the overall proportion of *An*. *arabiensis* between 2011 (84%) and the following years from 2012 to 2015 (53–68%) (Jonckheere-Terstra test statistic or *JT* = 660, *P* = 0.0028). The proportion of *An*. *coluzzii* increased from 11% in 2011 to 25–33% during the following years (*JT* = 622, *P* = 0.017), while the frequencies of *An*. *gambiae* which was 1–5% between 2011 and 2013 changed to 13–14% in 2014–2015.

### Spatio-temporal distribution of deltamethrin resistance

A total of 100 susceptibility tests were conducted during five years from 2011 to 2015, with 11, 534 mosquitoes exposed to 0.05% deltamethrin and 4, 039 control mosquitoes (Table 2). The numbers of the surveys conducted in each study site and the status of deltamethrin resistance in tested *An*. *gambiae s*.*l*. populations from 211 to 2015 are provided in Table 3. Of the 24 localities retained for the study, yearly screening of mosquito susceptibility during the five-year study period was conducted 5 times in 11 sites; 4 times in 10 sites and 1–2 times in the remaining 2 sites. Apart from 2011 when only 13 populations were tested, the number of populations surveyed each year varied between 19 and 23 depending on the sample size of larvae collected from field breeding sites.

**Table 3:** Layout of the monitoring and status of deltamethrin resistance in tested *An*. *gambiae s*.*l*. populations from 211 to 2015.

Health district	Locality	Mortality rates_(Resistance status)_				
		Nov. 2011	Oct. 2012	Oct. 2013	Oct. 2014	Oct. 2015
**Garoua**	Kanadi II	51.0_(R)_	63.0_(R)_	27.0_(R)_	40.0_(R)_	35.0_(R)_
	Kanadi I	ND	86.1_(R)_	85.6_(R)_	47.0_(R)_	61.9_(R)_
	Ouro housso	68.0_(R)_	46.2_(R)_	45.6_(R)_	32.0_(R)_	37.7_(R)_
	Lainde II	ND	75.0_(R)_	72.8_(R)_	48.0_(R)_	25.00_(R)_
	Djamboutou II	89.50_(R)_	70.4_(R)_	54.4_(R)_	69.0_(R)_	61.4_(R)_
	Plateau	ND	65.0_(R)_	42.2_(R)_	52.0_(R)_	44.6_(R)_
	Ouro lawane	ND	56,9_(R)_	64.4_(R)_	53_(R)_	24,4_(R)_
	Mbilga	ND	95.5_(PR)_	86.0_(R)_	67.0_(R)_	88.9_(R)_
	Kollere	22.0_(R)_	54.8_(R)_	43.0_(R)_	45_(R)_	39.0_(R)_
	Ouro garga	99.0_(S)_	76.5_(R)_	75.5_(R)_	77.0_(R)_	75.0_(R)_
**Pitoa**	Guizigare*	59.1_(R)_	63.0_(R)_	74.3_(R)_	64.2_(R)_	63.0_(R)_
	Pene	ND	90.8_(PR)_	74.5_(R)_	93.2_(PR)_	38.5_(R)_
	Boulgou*	ND	97.6_(PR)_	87.7_(R)_	98.7_(S)_	93.0_(PR)_
	Nassarao-be	ND	95.3_(PR)_	84.3_(R)_	95.7_(PR)_	75.5_(R)_
	Lombou	91.2_(PR)_	94.2_(PR)_	70.2_(R)_	93.7_(PR)_	51.1_(R)_
	Be-centre	78.9_(R)_	90.3_(PR)_	85.5_(R)_	74.0_(R)_	83.2_(R)_
	Mbolom	82.0_(R)_	89.6_(R)_	64.7_(R)_	UN	46.60_(R)_
**Mayo Oulo**	Mayo oulo	92.5_(PR)_	93.0_(PR)_	95.8_(PR)_	69.0_(R)_	83.5_(R)_
	Doumou	ND	ND	81.7_(R)_	49.0_(R)_	64.50_(R)_
	Matra	ND	ND	ND	86.0_(R)_	ND
	Maboni	ND	ND	80.0_(R)_	61.0_(R)_	ND
	Dourbeye*	82.5_(R)_	98.6_(S)_	85.9_(R)_	75.0_(R)_	78.9_(R)_
	Bossoum*	79.0_(R)_	UN	100_(S)_	81.0_(R)_	75.3_(R)_
	Bala	84.6_(R)_	100_(S)_	90.00_(PR)_	86.0_(R)_	81.6_(R)_
**Total**	**24**	**13**	**19**	**23**	**23**	**22**

Resistance_(R)_: Mortality rates < 90%

Probable resistance_(PR)_: 90% ≥ Mortality rates ≤ 98%

Susceptible_(S)_: Mortality rates > 98%

*Sites displaying sporadic variations of resistance status

ND: No data

Oct.: October; Nov.: November

**Fig 4:**
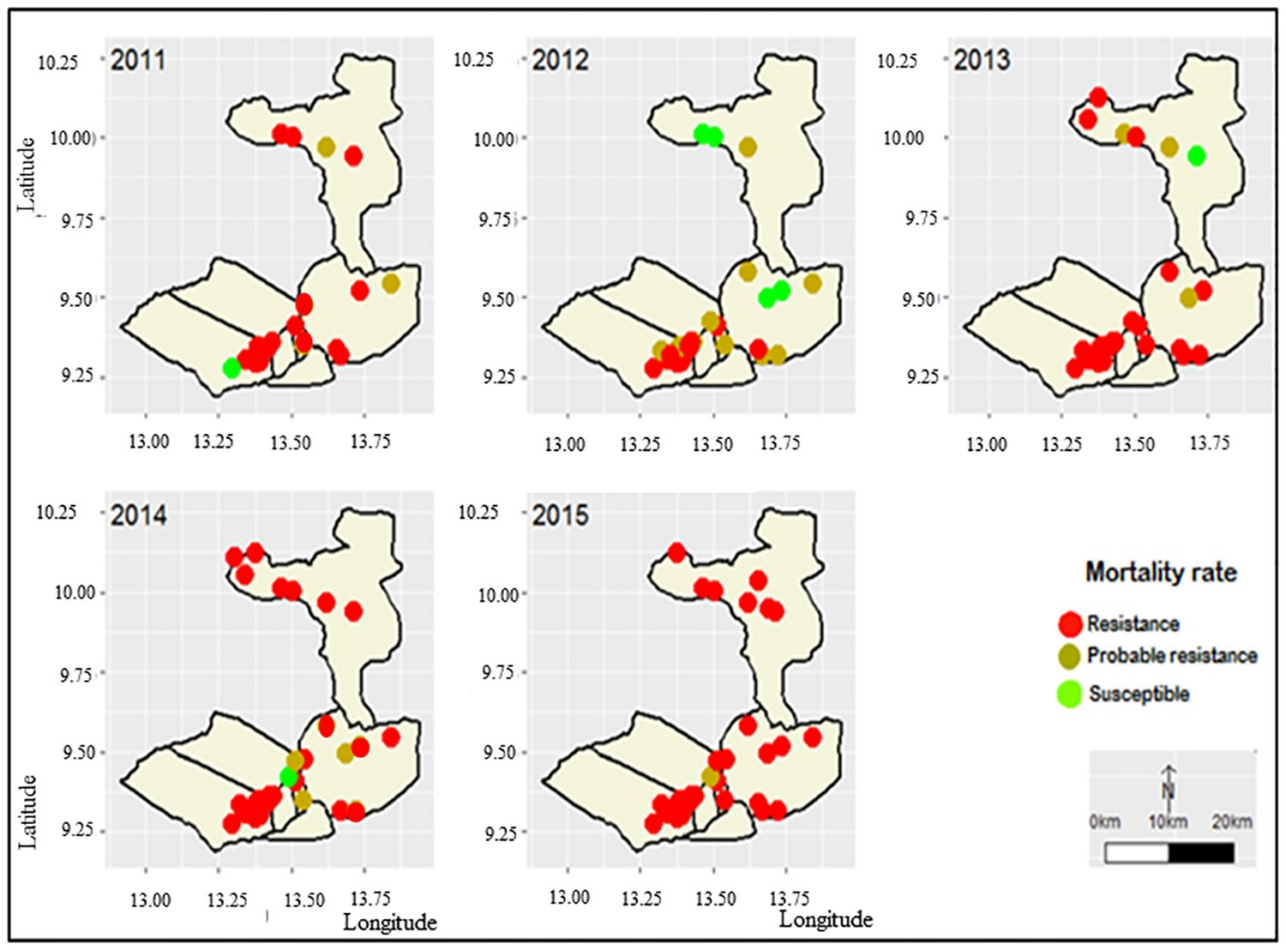
Spatial and temporal distribution of deltamethrin resistance in the study sites (2011–2015).

The geospatial pattern of *An*. *gambiae s*.*l*. resistance to deltamethrin in the prospected localities based on the WHO’s classification is presented in Fig 4. In 2011, only one out of the 13 tested *An*. *gambiae s*.*l*. populations was found susceptible to deltamethrin (Ouro-garga; Garoua HD), while two populations exhibited possible resistance (Mayo-Oulo in the Mayo Oulo HD; and Loumbou in the Pitoa HD). Ten populations displayed resistance, with different ranges of mortality rates according to the HDs; i.e. 22–89.5%, 59–82% and 79–85% in the Garoua, Pitoa and Mayou Oulo HDs respectively.

Afterward, the status of deltamethrin resistance was variable from year to year in 11 localities including 2 localities of the Garoua HD (Mbilga and Ouro Garga), 5 localities of the Pitoa HD (Pene, Boulgou, Nassarao-Be, Lombou, and Be-Centre) and 4 localities of the Mayo Oulo HD (Mayo Oulo, Dourbeye, Bossoum and Bala). In 7 of the 11 localities, the resistance status shifted from susceptible/possible resistance in 2012 (>90%) to confirmed resistance in 2013 (39–89% mortality rates), suggesting a broad evolvement of resistance at that period. In Ouro Garga (Garoua HD), resistance appeared earlier in 2012. In Bossoum (Mayo Oulo HD) the three resistance status (susceptible/possible resistance/resistance) were alternatively recorded from year to year. In most of the localities of the Pitoa HD, the status of resistance varied between possible and confirmed resistance. The 13 remaining mosquito populations mostly in the Garoua HD were found resistant during the whole study period, with mortality rates however decreasing in 11 populations from 46–86% in 2011 to 24–62% in 2015. Lastly in Kollere (Garoua HD) and Guizigare (Pitoa HD), the mortality rates all over the study period remained low (22–55%) and moderate (59–74%) respectively.

**Fig 5:**
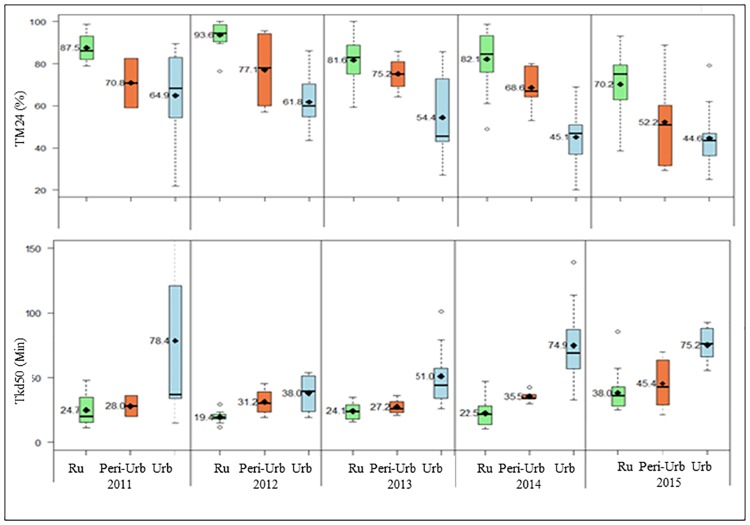
Spatial and temporal distribution of mortality rate (TM 24) and Tkd50 (knockdown times) according to the ecological features (2011–2015). Ru: rural settings; Peri-Urb: peri-urban settings; Urb: urban settings. Tkd50: time of knock-down for 50% tested mosquitoes; TM24: mortality rates 24 hours post exposure to insecticide.

Fig 5 shows differential profiles of resistance according to the ecological features, based on mortality rates (TM24) and knockdown times for 50% (Tkd50) of tested mosquitoes. During each survey-year, deltamethrin resistance was observed to gradually increase from the rural (93–70% mortality rates) to peri-urban (77–52% mortality rates) and urban settings (mortality = 64–44%) (*JT* = 5282, *P*< 0.0001). The decrease in mortality rates was associated with a significant increase in Tkd50 (*JT* = 5230, *P*< 0.0001) across the settings, and years (*JT* = 5636, *P*< 0.001), corresponding to 24–38 minutes, 27–45 minutes and 38–78 minutes in rural, peri-urban and urban settings respectively.

Spatial autocorrelation of mortality measurements in each of the survey-years from 2012 to 2015 was detected for all the localities in the study HDs; suggesting association in deltamethrin resistance between neighboring clusters and years of data collection (*P*< 0.0001), except at the beginning of the study in 2011 (*P* = 0.2296) (Table 4).

**Table 4:** Spatial autocorrelation between localities through the years.

Variable	Year	Moran’s Index		Standard deviation	*P*-value
		Observed	Expected		
**TM24**	2011	-0.0956331	-0.05263158	0.03579484	0.2296
	2012	-0.2512021	-0.03703704	0.02657929	<0.0001
	2013	-0.2253236	-0.03571429	0.02681102	<0.0001
	2014	-0.1495050	-0.02941176	0.02221648	<0.0001
	2015	-0.1654212	-0.02857143	0.02158000	<0.0001

TM24: mortality rate (%).

**Fig 6:**
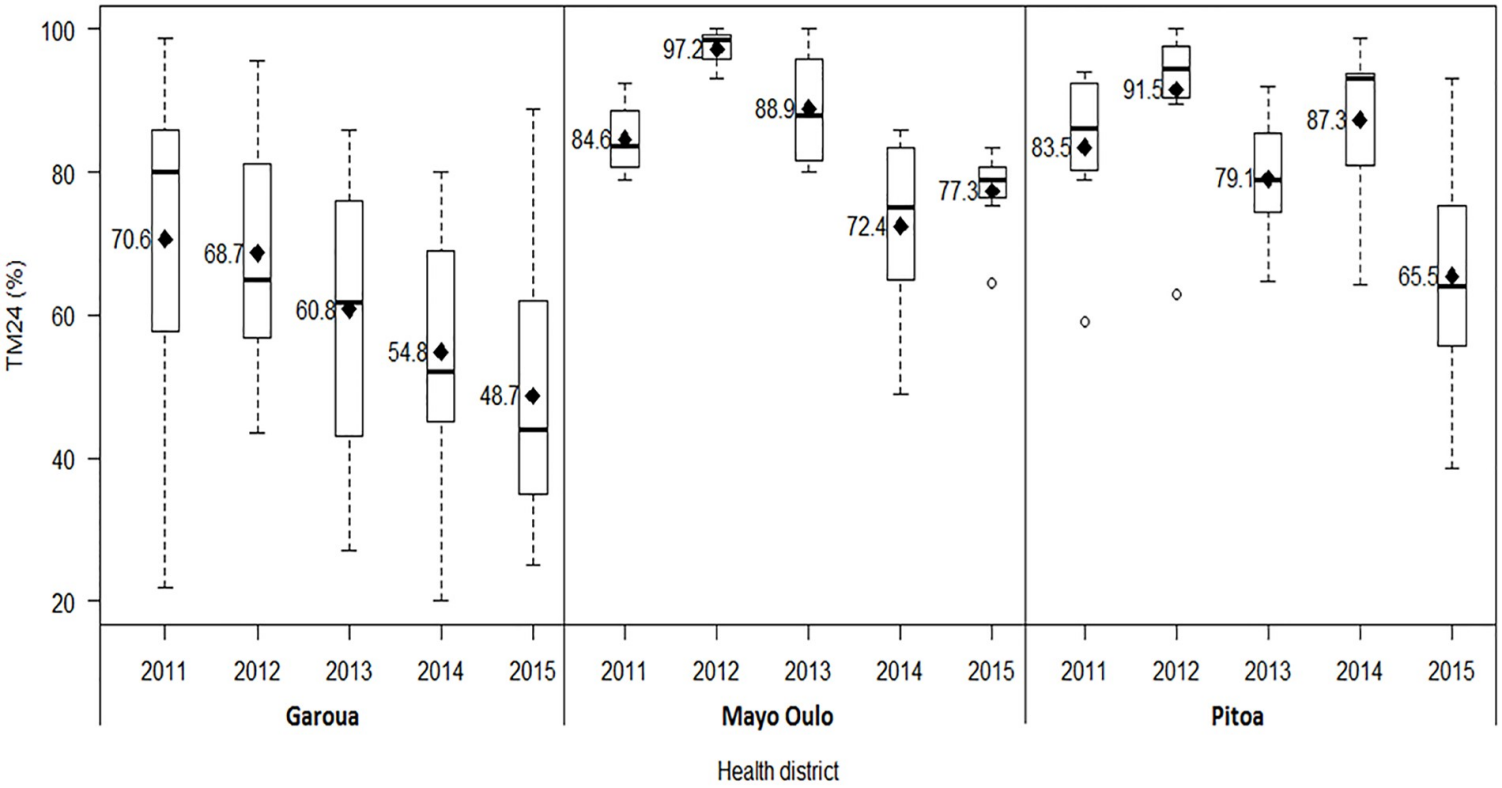
Temporal evolution of deltamethrin resistance in the study health districts (2011–2015). TM24: mortality rates 24 hours post exposure to insecticide.

When the yearly data from all the surveyed localities per HD were pooled, deltamethrin resistance frequencies were significantly increased (*P<0*.*005*) (Table 5, Fig 6). In the Garoua HD, the mortality rates gradually decreased from 70% in 2011 to approximately 50% in 2015. However in the Pitoa and Mayou Oulo HDs, the decrease of mortality rates over the years was nonlinear. The highest mortalities in these HDs were recorded in 2012 (91–97%) and the lowest in 2014–2015 (65–72%).

**Table 5:** Trends of overall district mortality rates from 2011 to 2015.

HD	2011 Mortality (95% CI)	2012 Mortality (95% CI)	2013 Mortality (95% CI)	2014 Mortality (95% CI)	2015 Mortality (95% CI)	*P*-value
**Garoua**	70.6 (55.9–85.3)	68.7 (60.3–77.1)	60.8 (51.0–70.6)	54.8 (46.4–63.3)	48.7 (40.2–57.3)	0.00015
**Pitoa**	83.5 (75.2–91.6)	91.5 (84.3–96.5)	79.1 (73.8–84.5)	87.3 (80.7–92.8)	65.5 (57.3–73.6)	0.0016
**Mayo Oulo**	84.6 (79.6–89.5)	97.2 (93.8–100.0)	88.9 (83.1–94.7)	72.4 (63.0–82.0)	77.3 (73.1–81.6)	0.0041

HD: Health District

Although the profiles of resistance were variable from one HD to another (*AD* = 21.83, *P*< 0.0001), a marked resistance increase was observed over the five years when considering the three HDs together (*JT = 5638*, *P<0*.*001*).

### Spatio-temporal evolution of *Kdr* 1014 allelic frequencies in *Anopheles gambiae s*.*l*.

Genotyping for the *Kdr* 1014 mutations was successfully performed on 2,077 mosquito specimens from 12 locations across the three HDs, including 913 specimens from the Garoua HD, 580 from the Pitoa HD and 584 from the Mayo Oulo HD (Table 2).

Both *Kdr* L1014F and L1014S alleles, as well as the wild type L1014L allele were found in *An*. *coluzzii* and *An*. *arabiensis*, while only the *Kdr* L1014F and the wild type L1014L alleles were found in *An*. *gambiae*. The *Kdr* L1014F allele was mostly carried by the *An*. *coluzzii* (64.87%), followed by *An*. *gambiae* (54.30%) and *An*. *arabiensis* (17.59%), while the *Kdr* L1014S occurred only in *An*. *coluzzii* and *An*. *arabiensis* at very low frequencies. The *Kdr* L1014F allele was widespread, with a higher frequency (31.5%) compared to the *Kdr* L1014S allele (2%).

**Fig 7:**
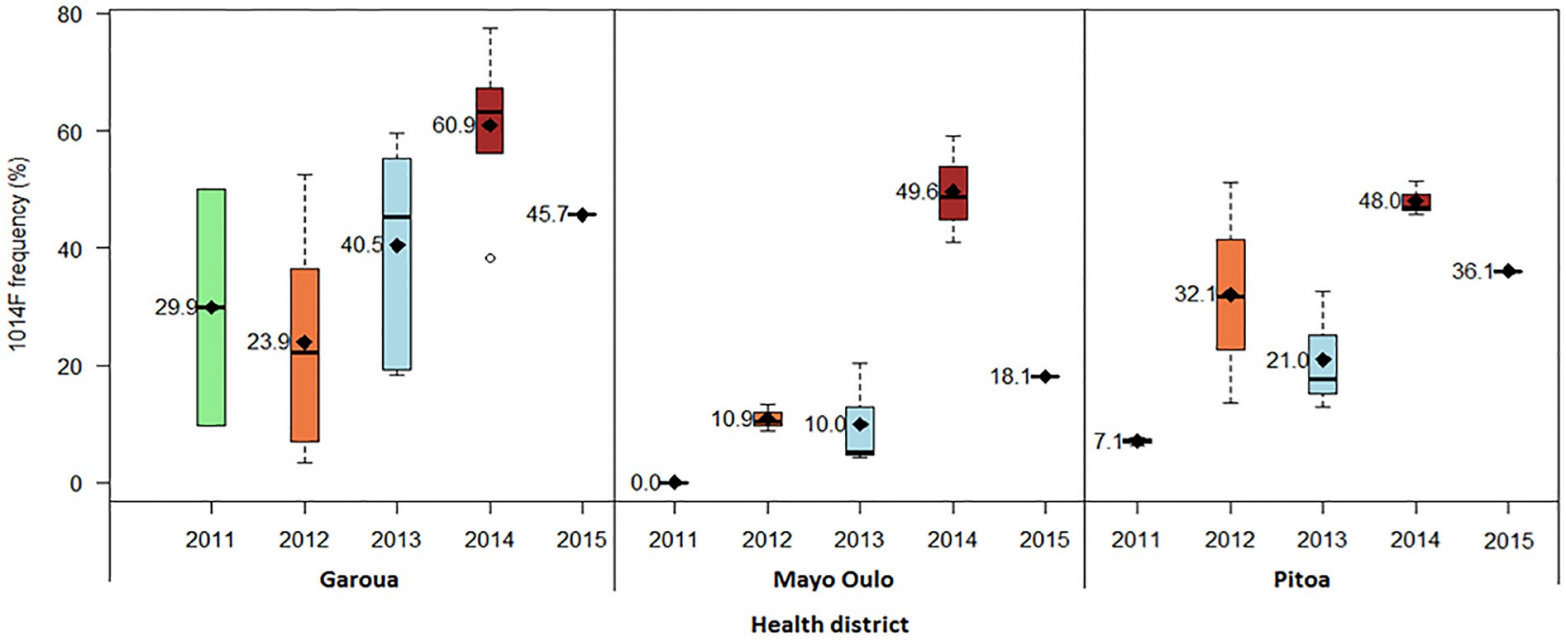
Variations in frequencies of knockdown resistance (*Kdr* L1014F) allele in *An*. *gambiae s*.*l*. populations (2011–2015).

There were marked differences in *Kdr* L1014F frequency between HDs (*AD* = 5.175, *P* = 0.01431), associated with an increase over the years (*JT* = 620, *P* = <0.001); the highest frequencies being recorded in the Garoua HD (Fig 7).

### Multiple factor analysis of resistance variations over the years

**Fig 8:**
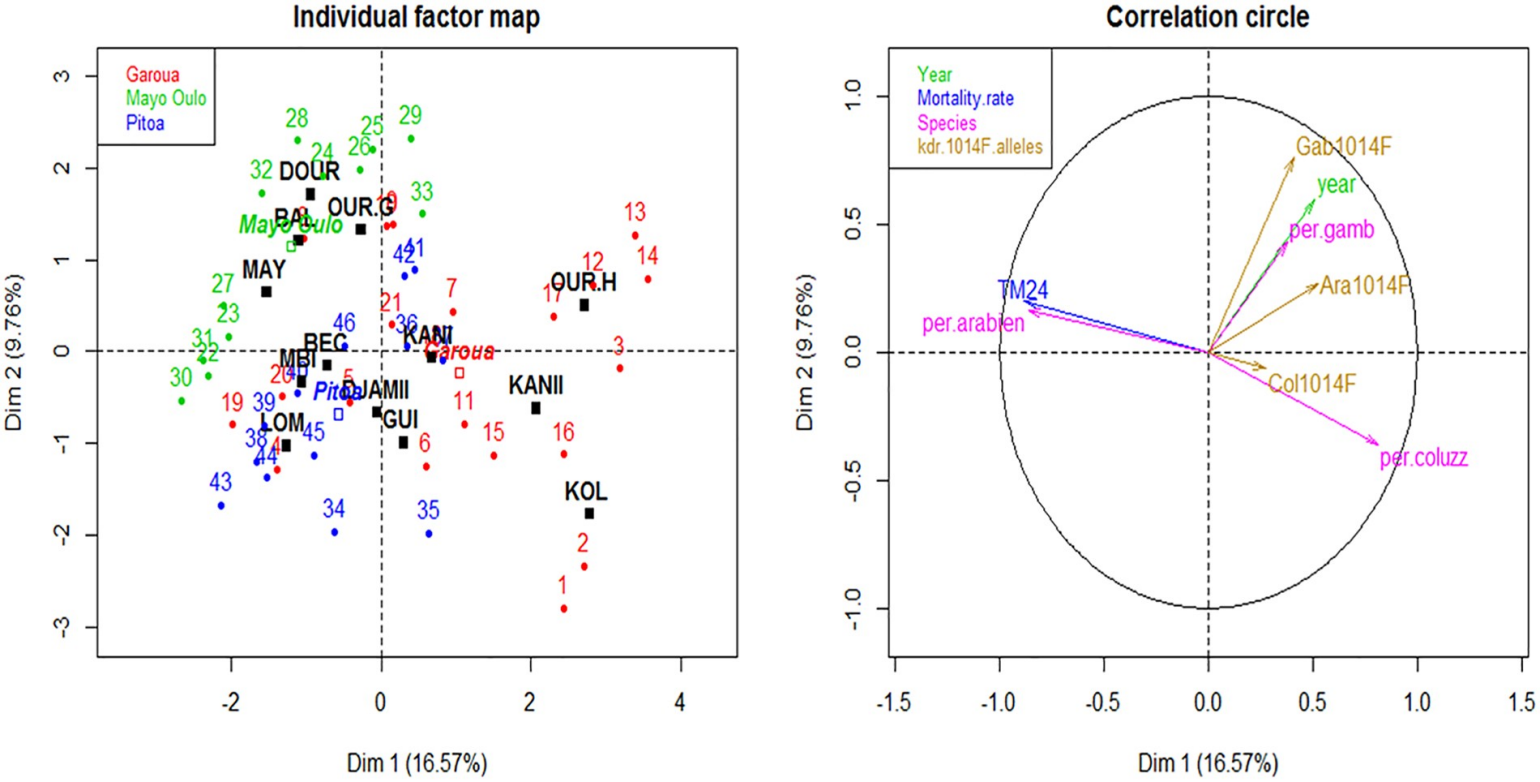
Multiple factor analysis (MFA): Representation of the individuals (localities) and variables on the first plane. Individual factor map showing the interaction between localities and years, coloured according to health districts (left). Correlation circle (right) is for the following sets of quantitative data: mortality rates, *Kdr* L1014F allele, species composition, and year. Health district was added as a supplementary variable. The analysis on the sets of data over the 5 years study period were done in localities were all the sets of data were available. TM24: TM24: mortality rates 24 hours post exposure to insecticides; per.arbien: percentage of *An*. *arabiensis*; per coluzz:percentage of *An*. *coluzzii*; per.gamb: percentage of *An*. *gambiae*: col1014F: allelic frequency of *Kdr* L1014F in *An*. *coluzzii*; Col L1014F: allelic frequency of *Kdr* L1014F in *An*. *coluzzii*; Ara L1014F: allelic frequency of *Kdr* L1014F in *An*. *arabiensis*; Gab 1014F: allelic frequency of *Kdr* L1014F in *An*. *gambiae*.

Multiple factor analysis of the data on *An*. *gambiae s*.*l*. mortality rates to deltamethrin, species distribution (*An*. *arabiensis*, *An*. *coluzzii*, *An*. *gambiae*) and the *Kdr* L1014F allelic frequencies over the 5 years study period were done for 12 localities were all the three data sets were available. The graphs of localities (individual factor map) and variables (correlation circle) show the relationship between localities, the quality of the representation of the localities, as well as the correlation between localities and the dimensions (Fig 8).

Generally, localities (and even their interaction with years) from the same HD were closer to each other, and were quite separated from those belonging to other HDs. The correlation circle shows a strong positive linear correlation between the mortality rates (TM24) and occurrence of *An*. *arabiensis*. Conversely, a strong negative correlation was observed between TM24 and proportion of *An*. *coluzzii*, and a weak negative correlation between TM24 and the proportion of *An*. *gambiae*. Also, there was a negative correlation between TM24 and the frequencies of *Kdr* L104F allele in *An*. *coluzzii* (Col1014F) and *An*. *arabiensis* (Ara1014F) respectively, with a weaker negative correlation between TM24 and the frequencies of *Kdr* L104F allele in *An*. *gambiae (*Gab1014F). TM24 was also weakly negatively related to year. The frequency of variables Col1014F (*Kdr* L104F frequencies in *An*. *coluzzii*), followed by per.gamb (proportion of *An*. *gambiae*) and Ara1014F (*Kdr* L104F frequencies in *An*. *arabiensis*) contributed less to the construction of the first and second dimensions.

## Discussion

Pyrethroid resistance in malaria vectors belonging to the *Anopheles gambiae* complex has become a major concern for malaria control in Cameroon, since it was first reported in 2003 [34]. So far, the trend of insecticide resistance was monitored through small-scale surveys or through retrospective and published data collected across the country Regions [19, 21, 35]. It is noteworthy that this study is the very first large scale 5-year longitudinal survey of insecticide resistance in malaria vectors from Cameroon. Data collected from 2011 to 2015 in 24 locations revealed a significant decrease in *An*. *gambaie s*.*l*. mortality rates to deltamethrin, suggesting increased frequency of resistance to this pyrethroid insecticide. However, there was a considerable heterogeneity in the resistance levels between localities and survey years. Such heterogeneity has also been reported in previous studies on spatial and temporal trends of insecticide resistance in field mosquito populations [36–39]. Considering the fact that pyrethroid insecticides have been commonly used in a number of the study sites and LLINs were distributed two times across the HDs at universal coverage in 2010 and 2015 in the study HDs, field populations of *An*. *gambiae s*.*l*. might be exposed to different patterns of selection pressure from one locality to another and from one year to another. Also, several other factors might influence the status of insecticide resistance, including the number of genes interacting to produce the phenotype of resistance, the dominance relationship of the alleles as well as the size and proportion of the population affected by insecticide treatments as suggested by Chareonviriyaphap et al. [40]. Furthermore, there is mounting evidence that when the level of resistance is high, mosquito mortality rates may not reflect changes in population resistance [41]. In this longitudinal study, the number of populations tested each year varied from 13 to 23, depending on the productivity of larval breeding sites. Some susceptibility test data were excluded from the analysis because fewer than 40 mosquitoes were tested. From the analyzed data, 10 out of the 13 populations tested at the beginning of the study in 2011 were found resistant to deltamethrin and the recorded mosquito mortality rates (60–90% in most of samples) revealed moderate to low levels of resistance in 7 of these populations, according to the stratification of Strode et al. [41] (Moderate: 25–80% mortality rate and < 25% *kdr* frequency; Low: >80% mortality rate and < 25% *kdr* frequency). During the 5 consecutive years of the study, the decrease of mortality rates alongside increase in knockdown times and the frequencies of *kdr* L1014F allele suggests significant changes toward high resistance as classified by Strode et al. [41] (<25% mortality rates, < 25% or >80% *kdr* frequency) in the three study HDs. This spatial and temporal development of deltamethrin resistance in *An*. *gambiae s*.*l*. could be linked to combined effects of environmental factors such as insecticide use in agriculture and in domestic hygiene tools such as ITNs, aerosol insecticide sprays, vaporizing mats, and mosquito coils [35, 42], as well as the factors inherent to the genetics and biology of the target vector species such as the mutations, migration and selection of resistance mechanisms [23]. Indeed, the extensive use of insecticides in both public health and cotton cultivation has been shown to affect the susceptibility of local malaria vector populations [35]. Among the 24 surveyed settings, resistance was more frequent in urban settings than in peri-urban and rural cultivated settings. In urban settings including most of the study sites of the Graoua HD, the selection pressure might have originated mainly from LLINs which was the main insecticide based mosquito control intervention during the study period. In these areas, *An*. *arabiensis* occurred in the same proportions as the more anthropophagic sibling species *An*. *coluzzii*. The latter species would mostly get into contact with insecticide treated substrates in an attempt to acquire human blood meals in the face of insecticide treatment [43].

The multifactorial analysis revealed a strong positive linear correlation between the mortality rates to deltamethrin and occurrence of *An*. *arabiensis* conversely to a strong negative correlation between the mortality rates and the proportions of *An*. *coluzzii*, and the proportion of *An*. *gambiae*. Also, there was a negative correlation between mortality rates and the frequencies of *Kdr* L104F allele in *An*. *coluzzii* and *An*. *arabiensis* respectively. These data suggest that the increase in the frequencies *An*. *coluzzii*, *An*. *gambiae* and the *Kdr* L1014 mutations led to the increase of deltamethrin resistance in the study areas. In rural areas including most of the study sites in the Mayo Oulo and Pitoa HDs where *An*. *arabiensis* was the predominant species, the high plasticity of biting habit between human and alternative hosts including livestock might reduce the frequency of its contact with treated substrates and subsequent exposure to insecticide selection pressure. These could explain the positive correlation of *An*. *arabiensis* frequencies with susceptibility to deltamethrin. The differential responses of vector species to interventions might have influenced the distribution of pyrethroid resistance across the country, and impacted on the dynamics of malaria transmission as reported in Burkina Faso [44].

The current study update the information on the distribution of members of the *An*. *gambiae* complex across the three study HDs and distribution of two important target-site resistance alleles (*Kdr* L1014F and L1014S) in these populations. Overall, *An*. *arabiensis* appears to be the predominant species in the study areas, followed by *An*. *coluzzii* and *An*. *gambiae*. The distribution of *An*. *arabiensis*, *An*. *coluzzii* and *An*. *gambiae* in the study sites is consistent with previous work carried out in North Cameroon [34, 35, 45–47]. *An*. *arabiensis* is known to colonize tropical areas from West to East Africa characterized by an accentuated drought of precipitations; meanwhile *An*. *coluzzii* and *An*. *gambiae* are highly adaptive malaria vectors colonizing both tropical and equatorial areas [48]. The distribution of these two species is associated with many factors such as the quality of the water [49], climate, vegetation, degree of urbanization [47] and insecticide pressure [42]. Accordingly, data from this study revealed increasing prevalence of *An*. *coluzzii* in surveyed locations, suggesting its progressive adaptation and dispersion in the north region of Cameroon. In addition, biotic interactions between *An*. *coluzzii* and *An*. *gambiae* occurring at larval and adult stages such as competition, predation and parasitism can further determine the structure of the species populations and have an impact on the balance and local distribution of species as reported in Burkina Faso [50, 51]. The urbanization gradient of the three surveyed HD could also explain the increase of *An*. *coluzzii* in collected samples. The ability of *An*. *coluzzii* to adapt in polluted breeding sites with predominance of organic matters in the urban agglomerations of Cameroon has been highlighted by Tene Fossog *et al*. [52]. Accordingly and also due to high altitude on hills (≈ 470 m), *An*. *coluzzii* was counterbalanced by *An*. *gambiae* in the Mayo Oulo rural HD, *An*. *coluzzii* being mostly adapted to altitudes ≤430 m [53].

On the other hand, the resistance phenotype was associated with an increase of knockdown times, suggesting the *Kdr* 1014 mutations among the mechanisms involved in deltamethrin resistance. Indeed, both L1014F and L1014S *Kdr* alleles were evidenced in *An*. *coluzzii* as well as in *An*. *arabiensis*, although L1014F allele occurred at a much higher frequency in *An*. *coluzzii* and *An*. *gambiae* than in *An*. *arabiensis*, in agreement with the previous reports [54–59]. To our knowledge, this is the first report of *kdr* L1014F allele at frequencies up to 65% in *An*. *coluzzii*, 54% *An*. *gambiae* and 18% *An*. *arabiensis* from North Cameroon and the first report of *Kdr* L1014S (2% frequency) in *An*. *coluzzii* and *An*. *arabiensis* from the same Region. The co-occurrence of both mutations in *An*. *coluzzii* and *An*. *gambiae* has widely been reported in the southern equatorial and Littoral Regions of Cameroon [21], but not in *An*. *arabiensis* which is the predominant malaria vector species in the Northern savanna Region. From 2011 to 2015, different trends of *kdr* L1014F frequencies were recorded in the three HDs, with variations from year to year. But in general, higher frequencies were recorded in 2014 (48–61%), followed by a decrease in 2015 (18–46%), versus 0–30% in 2011.

The reasons for these fluctuations are not clear, but the distribution of *kdr* 1014 mutations is known to differ among species of the *An*. *gambiae* complex including *An*. *gambiae*, *An*. *coluzzii* and *An*. *arabiensis* [60]. Many studies reported the kdr L1014F and L1014S mutations at high frequency within *An*. *gambiae* and *An*. *coluzzii* populations from the southern equatorial and littoral regions of Cameroon [21]. Over recent years, the frequencies of these mutations in specific settings significantly increased within both *An*. *coluzzii* and *An*. *gambiae* [21]. But very few was known about the prevalence and the spatial and temporal distribution of the *Kdr* L1014F and L1014S mutations in species of the *An*. *gambiae* complex in North Cameroon. Previous studies in Pitoa recorded the *Kdr* L1014F allele only in *An*. *coluzzii* at 34% frequency, and only in the heterozygous state. The *Kdr L1014S* allele was not found in any of the tested *An*. *arabiensis* nor in *An*. *coluzzii* specimens [5]. Chouaibou et al. [35] reported both *kdr L1014F* and L1014S alleles at very low frequencies (1/45, 1.1%) in *An*. *gambiae s*.*s*. specimens from Pitoa. From that time until 2011, no *Kdr* L1014 mutation has been reported in *An*. *arabiensis* from North Cameroon. The present study reveals that the *Kdr* L1014F has since spread across the three study HDs and is now observed at relatively high and similar frequencies (20–65%) across the southern equatorial and northern savanna regions of Cameroon. These findings are of great significance, since the spread of these mutations, in addition to metabolic resistance already reported in North Cameroon [54] may lead to a drastic increase of the frequencies and intensity of insecticide resistance in local malaria vector populations. The intensity of deltamethrin resistance in field samples of *An*. *gambiae s*.*l*. from Pitoa was found to play a major role in the gradual decrease of LifeNet bio-efficacy after serial washing, emphasizing the importance of monitoring insecticide resistance and its impact on vector control efficacy [5].

In this study, the allelic frequencies of *Kdr* L1014F varied within the three species with particularly high frequencies in *An*. *coluzzii*. Similar trend was recently observed in Manoka and Youpwe in the littoral region of Cameroon where *An*. *coluzzii* is the main malaria vector [61]. The rapid diffusion of the *Kdr* L1014F mutation in *An*. *coluzzii* and *An*. *gambiae* in Cameroon was suggested to have occurred by mutual introgression and via independent mutation events [23]. The origin of the *Kdr* L1014S mutation *An*. *coluzzii* and *An*. *arabiensis* from North Cameroon could also follow the same way of dissemination. In addition, transport of humans and goods across the country could favor the dissemination of mosquitoes carrying resistance alleles from one region to another. Sequence analysis of the intron-1 of the voltage-gated sodium channel gene flanking the *Kdr* locus may confirm whether the mutations recorded in *An*. *arabiensis*, *An*. *gambiae* and *An*. *coluzzii* from North Cameroon have evolved as de novo mutations or introgression from one species to another. The phenotypic outcomes of the co-occurrence of both L1014F and L1014S *Kdr* alleles, together with enzyme detoxification need also to be further examined.

Prior to this study, increased activity of cytochrome P450s monooxygenases was evidenced in *An*. *gambiae s*.*l*. populations from North Cameroon, using microplate enzyme assays on individual mosquitoes. This phenomena was mainly attributed to the selection of metabolic genes through the use of different pesticides in crop protection [54]. Cytochrome P450s can mediate resistance to all classes of insecticides, increased enzyme activity can be brought about by gene amplification, upregulation, coding sequence mutations, or by a combination of above mentioned mechanisms. More precisely, metabolic genes including *CYP4G16*, superoxide dismutases *SOD2* and *SOD3*, glutathione S-transferase *GSTS1* and thioredoxin dependent peroxidase *TPX4* were found to be involved in the detoxification process of deltamethrin in wild populations of *An*. *arabiensis* from Pitoa [22]. Coupled with the increase in the *Kdr* L1014F frequencies recorded in the present study, there is evidence of multiple insecticide resistance mechanisms in surveyed *An*. *gambiae s*.*l*. populations. These data confirmed the occurrence of multiple insecticide resistance mechanisms previously reported in several *An*. *gambiae s*.*l*. populations from Cameroon [19]. In Niger, large-scale distribution of LLINs led to an increased frequency of *Kdr* L1014F [11]. The use of pyrethroid insecticides at the household level and in small vegetable cultivation areas in Mali has also been reported to drive the *Kdr* L1014F to a higher frequency [62]. At operational level, metabolic-based resistance mechanisms in *An*. *funestus* were directly incriminated to the failure of pyrethroid insecticides to control malaria in South Africa [63]. It is likely that the dynamics of the *Kdr* 1014F allele, together with metabolic-based resistance in the three species of the *An*. *gambiae* complex induce the decline of LLINs effectiveness in North Cameroon in agreement with Strode *et al*. [41]. Indeed, malaria transmission in the Garoua, Pitoa and Mayo Oulo HDs remains very high regardless the use of LLINs, and it is mainly led by species of the *An*. *gambiae* complex, followed by *An*. *funestus*, *An*. *rufipes*, *An*. *paludis* and *An*. *pharoensis* [64]. Although no evidence of an association between infection prevalence or clinical incidence of malaria with higher pyrethroid resistance in vectors has been demonstrated yet [65], the pattern of deltamethrin resistance and the persistence of malaria transmission in the study HDs call attention to the urgent need for a resistance management strategy to sustain malaria vector control in North Cameroon.

## Conclusion

This study demonstrated rapid increase of deltamethrin resistance in *An*. *gambiae s*.*l*. populations from North Cameroon, associated with increasing occurrence of *An*. *coluzzii* among the sibling species of the *An*. *gambiae* complex and spread of the *Kdr* L1014F resistance allele. These data are essential for the development of vector surveillance and insecticide resistance management strategies. Trials of innovative vector control tools such as new generations of LLINs that use other insecticide classes or synergists are urgently needed in these areas to ascertain epidemiological and entomological impact in comparison to pyrethroid LLINs.

## Supporting information

S1 Dataset Overall species distribution and *Kdr* L1014F allelic frequencies across the study sites of the Garoua, Pitoa and Mayo Oulo heath districts (2011–2015). TM24: TM24: mortality rates 24 hours post exposure to insecticides; per.arbien: percentage of *An*. *arabiensis*; per coluzz:percentage of *An*. *coluzzii*; per.gamb: percentage of *An*. *gambiae*: col1014F: allelic frequency of *Kdr* L1014F in *An*. *coluzzii*; Col L1014F: allelic frequency of *Kdr* L1014F in *An*. *coluzzii*; Ara L1014F: allelic frequency of *Kdr* L1014F in *An*. *arabiensis*; Gab 1014F: allelic frequency of *Kdr* L1014F in *An*. *gambiae*.
(XLSX)

S1 Table Breakdowns of *Kdr* L1014F allelic frequencies in *An*. *arabiensis* from the three study Health districts (2011–2015). f (): allelic frequency (%); N_A_: number of analyzed *An*. *arabiensis* speciemens; p(HW): probability of the exact test for goodness of fit to Hardy- Weinberg equilibrium; in bold: Significant value (p(HW)<0.05, single test level); Fis is calculated according to Weir and Cockerham, 1984. Positive Fis indicates a deficit of heterozygotes and negative Fis indicates an excess of heterozygotes; ND: not determined because no polymorphism observed and/or N < 30.
(DOCX)

S2 Table Breakdowns of *Kdr* L1014F allelic frequencies in *An*. *coluzzii* from the three study health districts (2011–2015). f (): allelic frequency (%); N_c_: number of analyzed *An*. *coluzzii* specimens; p(HW): probability of the exact test for goodness of fit to Hardy- Weinberg equilibrium; in bold: Significant value (p(HW)<0.05, single test level); Fis is calculated according to Weir and Cockerham, 1984. Positive Fis indicates a deficit of heterozygotes and negative Fis indicates an excess of heterozygotes; ND: not determined because no polymorphism observed and/or N < 30.
(DOCX)

S3 Table Breakdowns of *Kdr* L1014F allelic frequencies in *An*. *gambiae* from the three study health districts (2011–2015). f (): allelic frequency (%); N_a_: number of analyzed *An*. *gambiae* specimens; p(HW): probability of the exact test for goodness of fit to Hardy- Weinberg equilibrium; in bold: Significant value (p(HW)<0.05, single test level); Fis is calculated according to Weir and Cockerham, 1984. Positive Fis indicates a deficit of heterozygotes and negative Fis indicates an excess of heterozygotes; ND: not determined because no polymorphism observed and/or N < 30.
(DOCX)

S1 Fig Data on *An*. *gambiae s*.*l*. susceptibility to 0.05% deltamethrin in the localities of the three health districts from 2011 to 2015. (A): Garoua health district; (B): Pitoa health district; (C): Mayo Oulo health district.
(DOCX)
