# Supplementary material for: Spatial and temporal development of deltamethrin resistance in malaria vectors of the Anopheles gambiae complex from North Cameroon
Source: PLoS One. 2019 Feb 19;14(2):e0212024. doi: 10.1371/journal.pone.0212024 (PMC6380565; doi:10.1371/journal.pone.0212024)
Supplement: S1 Table — f (): allelic frequency (%); NA: number of analyzed An. arabiensis speciemens; p(HW): probability of the exact test for goodness of fit to Hardy- Weinberg equilibrium; in bold: Significant value (p(HW)<0.05, single test level); Fis is calculated according to Weir and Cockerham, 1984. Positive Fis indicates a deficit of heterozygotes and negative Fis indicates an excess of heterozygotes; ND: not determined because no polymorphism observed and/or N < 30. (DOCX) [file pone.0212024.s002.docx]

**S1 Table.**

| **Years** | **Districts** | **Locality** | **N_A_** | **f (1014L)** | **f (1014F)** | **f (1014S)** | **Fis** | **p(HW)** |
| --- | --- | --- | --- | --- | --- | --- | --- | --- |
| 2011 | GAROUA | Kanadi II | 20 | 62,50 | 37,50 | 0 | +0,898 | **0,0000** |
|  |  | Djamboutou II | 34 | 100 | 0 | 0 | ND | - |
| 2012 | GAROUA | Ouro housso II | 35 | 77,14 | 22,86 | 0 | +0,684 | **0,0002** |
|  |  | Djamboutou II | 32 | 90,63 | 9,38 | 0 | +0,279 | 0,9882 |
|  |  | Kanadi I | 11 | 81,82 | 18,18 | 0 | +1,000 | **0,0067** |
|  |  | Mbilga | 40 | 96,25 | 3,75 | 0 | +0,661 | **0,0377** |
|  |  | Ouro garga | 43 | 100 | 0 | 0 | ND | - |
| 2013 | GAROUA | Kanadi II | 23 | 84,78 | 13,04 | 2,17 | +0,8417 | **0,0001** |
|  |  | Djamboutou II | 24 | 97,92 | 2,08 | 0 | ND | - |
|  |  | Ouro housso II | 19 | 76,32 | 23,68 | 0 | -0,2857 | 0,2902 |
|  |  | Ouro garga | 34 | 75,00 | 25,00 | 0 | +0,1519 | 0,9111 |
|  |  | Kollere | 11 | 90,91 | 9,09 | 0 | +1,1000 | **0,0472** |
|  |  | Mbilga | 24 | 87,50 | 12,50 | 0 | +1,0435 | **0,0001** |
| 2014 | GAROUA | Kanadi II | 28 | 39,29 | 60,71 | 0 | +0,2683 | 0,2325 |
|  |  | Djamboutou II | 14 | 57,14 | 42,86 | 0 | +0,7263 | **0,0092** |
|  |  | Ouro housso II | 16 | 18,75 | 81,25 | 0 | +0,2105 | 0,4324 |
|  |  | Ouro garga | 42 | 45,24 | 54,76 | 0 | +0,3379 | **0,0359** |
|  |  | Kollere | 9 | 55,56 | 44,44 | 0 | +0,5897 | 0,1715 |
|  |  | Mbilga | 30 | 56,67 | 43,33 | 0 | +0,3364 | 0,9878 |
| 2011 | PITOA | Lombou | 39 | 97,44 | 2,56 | 0 | +1,000 | **0,0123** |
|  |  | Be-centre | 41 | 95,12 | 4,88 | 0 | +1,000 | **0,0004** |
|  |  | Guizigare | 33 | 100 | 0 | 0 | ND | - |
| 2012 | PITOA | Lombou | 23 | 84,78 | 15,22 | 0 | -0,158 | 0,5832 |
|  |  | Be-centre | 30 | 96,67 | 3,33 | 0 | +1,000 | **0,0167** |
|  |  | Guizigare | 15 | 100 | 0 | 0 | ND | - |
| 2013 | PITOA | Lombou | 30 | 98,33 | 1,67 | 0 | ND | - |
|  |  | Be-centre | 38 | 96,05 | 3,95 | 0 | +0,6753 | **0,0407** |
|  |  | Guizigare | 23 | 97,83 | 2,17 | 0 | ND | - |
| 2014 | PITOA | Lombou | 23 | 71,74 | 28,26 | 0 | +0,2701 | 0,9650 |
|  |  | Be-centre | 20 | 95,00 | 5,00 | 0 | +1,0000 | **0,0251** |
|  |  | Guizigare | 20 | 25,00 | 75,00 | 0 | +0,2245 | 0,5417 |
| 2011 | MAYO OULO | Mayo oulo | 50 | 100 | 0 | 0 | ND | - |
|  |  | Bala | 40 | 100 | 0 | 0 | ND | - |
| 2012 | MAYO OULO | Mayo oulo | 43 | 89,53 | 10,47 | 0 | -0,105 | 0,6265 |
|  |  | Bala | 43 | 90,70 | 9,30 | 0 | +0,184 | 0,9697 |
|  |  | Dourbeye | 40 | 90,00 | 10,00 | 0 | +0,179 | 0,9674 |
| 2013 | MAYO OULO | Mayo oulo | 45 | 100 | 0 | 0 | ND | - |
|  |  | Bala | 41 | 95,12 | 4,88 | 0 | -0,0394 | 0,9242 |
|  |  | Dourbeye | 46 | 98,91 | 1,09 | 0 | ND | - |
| 2014 | MAYO OULO | Mayo oulo | 15 | 90,00 | 10,00 | 0 | +0,6500 | 0,1039 |
|  |  | Bala | 34 | 85,29 | 14,71 | 0 | +0,5417 | **0,0106** |
|  |  | Dourbeye | 24 | 85,42 | 14,58 | 0 | +0,8392 | **0,0014** |
