# Supplementary material for: Spatial and temporal development of deltamethrin resistance in malaria vectors of the Anopheles gambiae complex from North Cameroon
Source: PLoS One. 2019 Feb 19;14(2):e0212024. doi: 10.1371/journal.pone.0212024 (PMC6380565; doi:10.1371/journal.pone.0212024)
Supplement: S2 Table — f (): allelic frequency (%); Nc: number of analyzed An. coluzzii specimens; p(HW): probability of the exact test for goodness of fit to Hardy- Weinberg equilibrium; in bold: Significant value (p(HW)<0.05, single test level); Fis is calculated according to Weir and Cockerham, 1984. Positive Fis indicates a deficit of heterozygotes and negative Fis indicates an excess of heterozygotes; ND: not determined because no polymorphism observed and/or N < 30. (DOCX) [file pone.0212024.s003.docx]

**S2 Table.**

| **Years** | **Districts** | **Locality** | **N_C_** | **f (1014L)** | **f (1014F)** | **f (1014S)** | **Fis** | **p(HW)** |
| --- | --- | --- | --- | --- | --- | --- | --- | --- |
| 2011 | GAROUA | Kanadi II | 18 | 38,89 | 61,11 | 0 | +0,093 | 1,000 |
|  |  | Djamboutou II | 4 | 12,50 | 87,50 | 0 | ND | - |
| 2012 | GAROUA | Ouro houssoII | 12 | 54,17 | 45,83 | 0 | +0,2029 | 0,9059 |
|  |  | Djamboutou II | 5 | 50,00 | 50,00 | 0 | -0,0909 | 0,7656 |
|  |  | Kanadi I | 25 | 36,00 | 64,00 | 0 | -0,3714 | 0,0756 |
|  |  | Kollere | 43 | 62,79 | 37,21 | 0 | +0,4126 | 0,9991 |
|  |  | Mbilga | 3 | 50,00 | 50,00 | 0 | -1,0000 | 0,3974 |
| 2013 | GAROUA | Kanadi II | 22 | 11,36 | 88,64 | 0 | -0,1053 | 1,0000 |
|  |  | Djamboutou II | 13 | 50,00 | 50,00 | 0 | +0,2683 | 0,9418 |
|  |  | Ouro housso II | 27 | 24,07 | 75,93 | 0 | -0,3000 | 0,2850 |
|  |  | Ouro garga | 11 | 13,64 | 86,36 | 0 | -0,1111 | 1,0000 |
|  |  | Kollere | 36 | 25,00 | 75,00 | 0 | -0,1715 | 0,4027 |
|  |  | Mbilga | 2 | 0 | 100 | 0 | ND | - |
| 2014 | GAROUA | Kanadi II | 10 | 50,00 | 50,00 | 0 | +0,2500 | 0,9291 |
|  |  | Djamboutou II | 12 | 37,50 | 62,50 | 0 | +0,5000 | 0,2018 |
|  |  | Ouro housso II | 16 | 34,38 | 65,63 | 0 | +0,0625 | 1,0000 |
|  |  | Ouro garga | 12 | 33,33 | 66,67 | 0 | -0,0820 | 0,6556 |
|  |  | Kollere | 21 | 26,19 | 73,81 | 0 | -0,0843 | 1,0000 |
|  |  | Mbilga | 20 | 70,00 | 30,00 | 0 | +0,7725 | **0,0012** |
| 2011 | PITOA | Lombou | 3 | 33,33 | 66,67 | 0 | -0,333 | 1,000 |
|  |  | Be-centre | 3 | 66,67 | 33,33 | 0 | +1,000 | 0,2042 |
|  |  | Guizigare | 5 | 40,00 | 60,00 | 0 | +0,273 | 1,000 |
| 2012 | PITOA | Lombou | 7 | 14,29 | 85,71 | 0 | +1,0000 | 0,0783 |
|  |  | Be-centre | 7 | 42,86 | 57,14 | 0 | +0,4783 | 0,4402 |
|  |  | Guizigare | 28 | 21,43 | 78,57 | 0 | +0,3793 | 0,0745 |
| 2013 | PITOA | Lombou | 7 | 14,29 | 85,71 | 0 | -0,0909 | 1,0000 |
|  |  | Be-centre | 5 | 20,00 | 80,00 | 0 | -0,1429 | 1,0000 |
|  |  | Guizigare | 9 | 33,33 | 66,67 | 0 | +0,0588 | 1,0000 |
| 2014 | PITOA | Lombou | 18 | 27,78 | 72,22 | 0 | -0,0794 | 1,0000 |
|  |  | Be-centre | 16 | 28,13 | 71,88 | 0 | -0,0500 | 1,0000 |
|  |  | Guizigare | 12 | 83,33 | 16,67 | 0 | -0,1579 | 0,7470 |
| 2012 | MAYO OULO | Bala | 2 | 0 | 100 | 0 | ND | - |
|  |  | Dourbeye | 5 | 100 | 0 | 0 | ND | - |
| 2013 | MAYO OULO | Bala | 1 | 0 | 100 | 0 | ND | - |
| 2014 | MAYO OULO | Bala | 8 | 6,25 | 93,75 | 0 | ND | - |
|  |  | Mayo oulo | 8 | 12,50 | 81,25 | 6,25 | +0,2821 | 0,2063 |
|  |  | Dourbeye | 12 | 0 | 100 | 0 | ND | - |
