# Supplementary material for: Spatial and temporal development of deltamethrin resistance in malaria vectors of the Anopheles gambiae complex from North Cameroon
Source: PLoS One. 2019 Feb 19;14(2):e0212024. doi: 10.1371/journal.pone.0212024 (PMC6380565; doi:10.1371/journal.pone.0212024)
Supplement: S3 Table — f (): allelic frequency (%); Na: number of analyzed An. gambiae specimens; p(HW): probability of the exact test for goodness of fit to Hardy- Weinberg equilibrium; in bold: Significant value (p(HW)<0.05, single test level); Fis is calculated according to Weir and Cockerham, 1984. Positive Fis indicates a deficit of heterozygotes and negative Fis indicates an excess of heterozygotes; ND: not determined because no polymorphism observed and/or N < 30. (DOCX) [file pone.0212024.s004.docx]

**S3 Table.**

| **Years** | **Districts** | **Locality** | **N_S_** | **f (1014L)** | **f (1014F)** | **f (1014S)** | **Fis** | **p(HW)** |
| --- | --- | --- | --- | --- | --- | --- | --- | --- |
| 2011 | GAROUA | Kanadi II | 8 | 43,75 | 56,25 | 0 | -0,207 | 1,000 |
|  |  | Djamboutou II | 3 | 83,33 | 16,67 | 0 | ND | - |
| 2012 | GAROUA | Djamboutou II | 1 | 50,00 | 50,00 | 0 | ND | - |
|  |  | Kanadi I | 4 | 25,00 | 75,00 | 0 | -0,2000 | 1,0000 |
|  |  | Kollere | 1 | 100 | 0 | 0 | ND | - |
|  |  | Ouro garga | 2 | 25,00 | 75,00 | 0 | ND | - |
| 2013 | GAROUA | Kanadi II | 2 | 75,00 | 25,00 | 0 | ND | - |
|  |  | Djamboutou II | 1 | 100 | 0 | 0 | ND | - |
|  |  | Ouro housso II | 1 | 0 | 100 | 0 | ND | - |
|  |  | Ouro garga | 2 | 25,00 | 75,00 | 0 | ND | - |
| 2014 | GAROUA | Kanadi II | 5 | 30,00 | 70,00 | 0 | +0,6000 | 0,3353 |
|  |  | Djamboutou II | 9 | 33,33 | 66,67 | 0 | +0,0588 | 1,0000 |
|  |  | Ouro housso II | 8 | 6,25 | 93,75 | 0 | ND | - |
|  |  | Ouro garga | 3 | 66,67 | 33,33 | 0 | -0,3333 | 0,7958 |
|  |  | Kollere | 2 | 0 | 100 | 0 | ND | - |
|  |  | Mbilga | 5 | 60,00 | 40,00 | 0 | +1,0000 | **0,0479** |
| 2011 | PITOA | Lombou | 1 | 100 | 0 | 0 | ND | - |
|  |  | Be-centre | 3 | 100 | 0 | 0 | ND | - |
|  |  | Guizigare | 1 | 100 | 0 | 0 | ND | - |
| 2013 | PITOA | Dourbeye | 2 | 0 | 100 | 0 | ND | - |
|  |  | Guizigare | 8 | 18,75 | 81,25 | 0 | -0,1667 | 1,0000 |
| 2014 | PITOA | Guizigare | 4 | 62,50 | 37,50 | 0 | +0,5714 | 0,4413 |
|  |  | Lombou | 7 | 57,14 | 42,86 | 0 | +0,4783 | 0,4402 |
|  |  | Be-centre | 12 | 20,83 | 79,17 | 0 | +0,2826 | 0,4094 |
| 2013 | MAYO OULO | Mayo oulo | 2 | 0 | 100 | 0 | ND | - |
|  |  | Bala | 7 | 0 | 100 | 0 | ND | - |
| 2014 | MAYO OULO | Mayo oulo | 21 | 14,29 | 85,71 | 0 | +0,2453 | 0,3394 |
|  |  | Bala | 8 | 0 | 100 | 0 | ND | - |
|  |  | Dourbeye | 4 | 0 | 100 | 0 | ND | - |
