# Supplementary material for: Spatial and temporal development of deltamethrin resistance in malaria vectors of the Anopheles gambiae complex from North Cameroon
Source: PLoS One. 2019 Feb 19;14(2):e0212024. doi: 10.1371/journal.pone.0212024 (PMC6380565; doi:10.1371/journal.pone.0212024)
Supplement: S1 Fig — (A): Garoua health district; (B): Pitoa health district; (C): Mayo Oulo health district. (DOCX) [file pone.0212024.s005.docx]

**A**

**B**

**C**

**S1 Fig.**
